# Notice of Gross’ System of Surgery

**Published:** 1859-10

**Authors:** 


*Gross' System of Surgery.* In two volumes, 8vo., of about 2400 pages. We have just time to announce the completion of this great work. We hope before the next issue to be able to present a more extended analysis. In the mean time we give the following letter, which we have just received from an eminent surgical friend:

"With pleasure we record the completion of this long-anticipated work. The reputation which the author has for many years sustained, both as a surgeon and as a writer, had prepared us to expect a treatise of great excellence and originality; but we confess we were by no means prepared for the work which is before us—the most complete treatise upon Surgery ever published, either in this or in any other country, and we might, perhaps, safely say, the most original. There is no subject belonging properly to surgery which has not received from the author a due share of attention; and if, in consequence, the work has acquired enormous dimensions, the blame must not be charged to Dr. Gross, but rather to that prodigious growth of the art of Surgery during the last half a century, which has rendered such labors necessary. But these two volumes are not equal in size to either the three volumes of Velpeau, published in this country with Notes by Townsend and Mott, or to the three volumes of Chelius, with Notes by South. Yet in completeness and in real practical merit, they are superior to either of the latter. Such will, probably, be the judgment of all who shall have an opportunity to compare these great works with each other. Neither Chelius or Velpeau have attempted to discuss the whole subject of surgery; yet they are each of them more voluminous than Gross. With the numerous smaller surgical treatises of the German, French, English and American writers, it is impossible to institute a comparison. They are relatively mere hand-books, or, as they are generally termed, "text-books," which were intended only to furnish the medical student with the outlines of the author's lectures, or which have been "compiled" by book-makers for the trade. These books have their value, and could not very well be spared, especially by the student of medicine; but Dr. Gross has supplied a want in surgical literature which has long been felt by practitioners: he has furnished us with a complete practical treatise upon Surgery in all of its departments. As Americans, we are proud of the achievement; and as surgeons, we are most sincerely thankful to him for his extraordinary labors in our behalf."

